# Supplementary material for: Overexpression of the Aldehyde Dehydrogenase Gene ZmALDH Confers Aluminum Tolerance in Arabidopsis thaliana
Source: Int J Mol Sci. 2022 Jan 1;23(1):477. doi: 10.3390/ijms23010477 (PMC8745680; doi:10.3390/ijms23010477)
Supplement: Supplementary file 1 [file ijms-23-00477-s001.zip › ijms-1501040-supplementary.pdf]

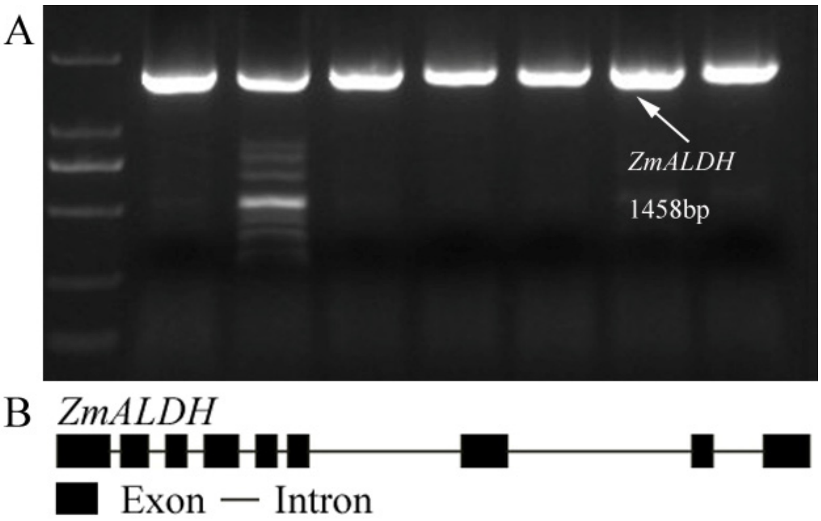

Fig. S1 Amplification of *ZmALDH* and gene structure

(A) PCR amplification of the *ZmALDH* gene. (B) Gene structure of *ZmALDH*.

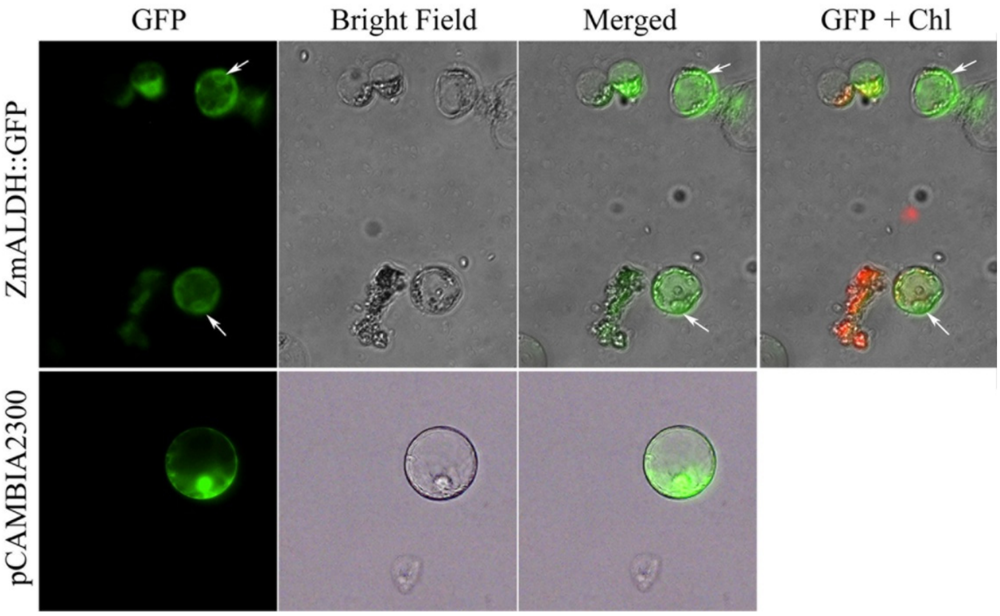

Fig. S2 Subcellular localization of ZmALDH in the protoplast of maize

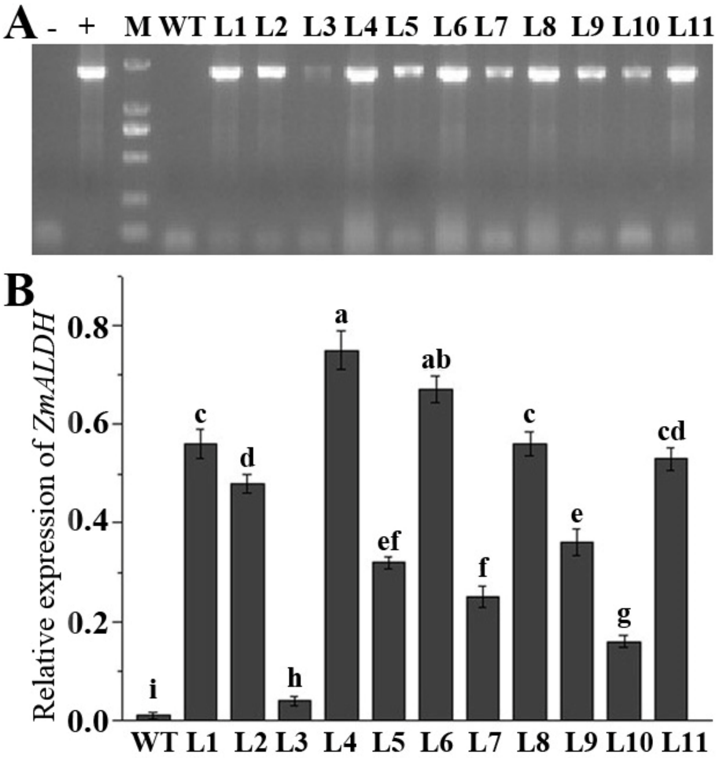

Fig. S3 Molecular analyses of *ZmALDH*-overexpressing *Arabidopsis*

Positive detection of *ZmALDH*-overexpressing plants by (**A**) PCR and (**B**) RT-PCR. Finally, two lines (L4 and L6) with the highest *ZmALDH* expression levels were identified. The *AtACT2* was used as the internal standard. Values are means ± SD (n = 20). Different letters indicate significant differences ($P < 0.01$) (Tukey's test).

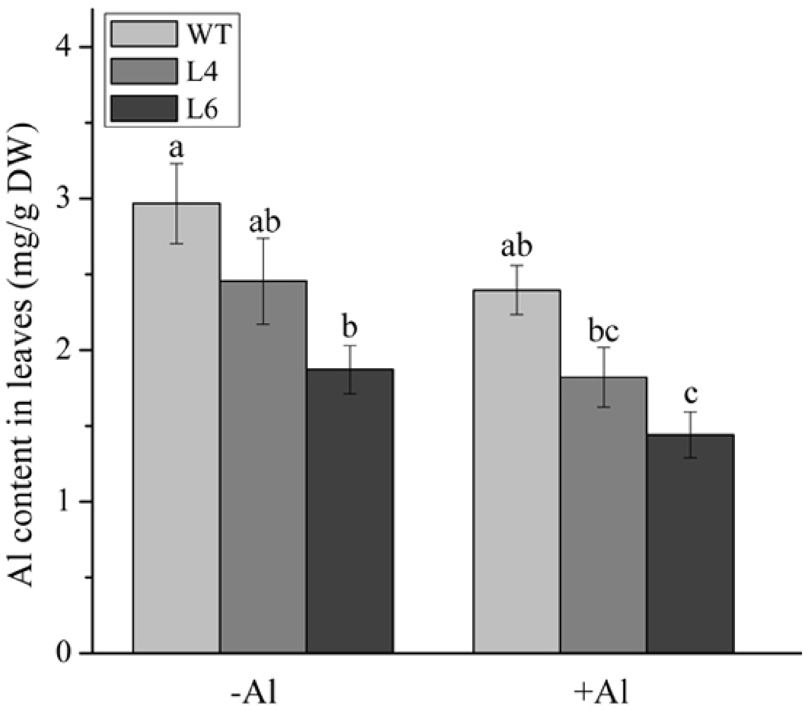

Fig. S4 Al contents in leaves in transgenic lines and wild-type plants

Al contents in wild-type and transgenic lines under normal conditions and Al stress for 12 h. Values represent mean ± SD ($n \geq 15$). Different letters indicate significant differences ($P < 0.01$) (Tukey's test).

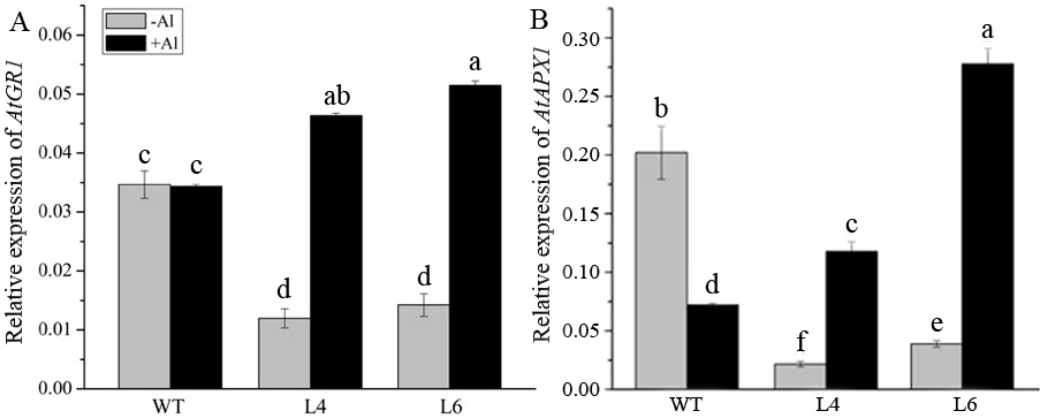

Fig. S5 Transcript levels of *AtGR1* and *AtAPX1* in wild-type and transgenic *Arabidopsis*

Expression analysis of (**A**) *AtGR1* and (**B**) *AtAPX1* in wild-type plants and transgenic lines after exposure to Al for 12 h. The *AtACT2* was used as the internal standard. Values are means ± SD ($n = 20$). Different letters indicate significant differences ($P < 0.01$) (Tukey's test).

Table S1 Basic information for *ZmALDH*

Gene name	Locus tag	Chr[a]	ORF length (bp)	Protein			Tm domains
				Length (No. of amino acids)	Mass (kDa)	pI	
ZmALDH	*Zm00001d017418*	5	1458	446	49.81	6.40	1

a: chromosome

Table S2 Prediction of the subcellular localization of ZmALDH

Program	Predicted site	Online linkage
YLoc	Cytoplasm	https://abi-services.informatik.uni-tuebingen.de/yloc/webloc.cgi?id=00c05db4c5a770a89dcfc66c3021ff64
Plant-mPLoc	Chloroplast	http://www.csbio.sjtu.edu.cn/cgi-bin/PlantmPLoc.cgi
BUSCA	cytoplasm	http://busca.biocomp.unibo.it/297a5a60-3d95-4504-a66b-ace67090c278/showresult/
WoLFPSORT	Cytoplasm<br>ER<br>Chloroplast<br>Nuclear	https://www.genscript.com/tools/wolf-psort/detail?file=2020/09/08/htdocs/results/159956565728765.detailed1.html#159956565728765

Table S3 Primers used in the study

Program	Forward	Reverse
ZmALDH for gene cloning	5'-TACGCAGATAGAGACCAGCAGC-3'	5'-ACTGTGTGTTGGGTGTGGGT-3'
ZmALDH for overexpression	5'-CGGGATCCATGGGGAGTGTACCGGAGGAG-3'	5'-GGAATTCTCATCTCCTGAGGCCGATGAG-3'
ZmALDH for subcellular localization	5'-GGGGTACCATGGGGAGTGTACCGGAGGAG-3'	5'-CGGGATCCTCTCCTGAGGCCGATGAGC-3'
ZmGAPDH for RT-PCR	5'-CCATCACTGCCACACAGAAAAC-3'	5'-AGGAACACGGAAGGACATACCAG-3'
AtACT2 for RT-PCR	5'-GCTGGATTCTGGTGATGGT-3'	5'-GCTCTGCTGTTGTGGTGAA-3'
AtPOD1 for RT-PCR	5'-GCAGCACACTTGCCATACAG-3'	5'-GTCCCATGCCGATGAGAACT-3'
AtAPX1 for *RT-PCR*	5'-AGTTGGATTTCGTGAGTTTTGGA-3'	5'-TCCTGCACTTCTCAACAGCC-3'
AtSOD1 for RT-PCR	5'-AACTGCCACCTTCACAATCACT-3'	5'-AGTTCATGGCCTCCCTTTCC-3'
AtCAT1 for *RT-PCR*	5'-CCGCAGTGGAATCTCTTCGT-3'	5'-GGAACCACAAGAGCAGGACA-3'
AtGR1 for RT-PCR	5'-TTCCTCCTCTCCGTATCTGTGT-3'	5'-GCAGACTCACACAAATCCAAACTTA-3'

Table S4 ALDH protein sequences used in the study

>ZmALDH

MGSVPEEKAKLGFGGLVGDLREVYESGRTQGLEWRQSQLRGLVRLLEEKEEEIFDVLHEDLGKHRGEAF
RDEAHTPLVAFPATALVVPEPLGVVLVFSCWNLPIGLALEPLSGALAAGNAVVVKPSELAPATSAFLAANIP
KYLDSKAVKVVEGGPEVGEKLMEHRWDKAAKHLTPVALELGSKCPCIVDWLDSDRDSQVAVNRIIGAKW
STCSGQACIAIDYLLVEEEFAPILIEMLKSTLERFFTKPEYMARILNEKQFQRLSGFLADRRVASSVVHGGHF
NPKTLSMEPTLLLNPPLDSDIMTEEIFGPLLPIITVKKIEDSIKFLRSKPKPLAIYAFTRNEKLKQRIIDETSSGS
ITFNDAIVQYGLDSIPFGGVGHSGFGQYHGKYSFDMFSHKKAVLKRSFLVEFMFRYPPWDETKIGMLRRV
YRFDYVSLFLALIGLRR

>OsALDH3-1

MEEKPQHGSLGGLVAGVREEYESGRTKELEWRKAQLGGLIRMITEEEDAIFDALHDDLGKHRVESFRDEV
GVLAKSVRNTLQNLKKWASPEKVDVPLISFPCNARVVPEPIGVVLIFSCWNLPIGLALEPLSGAIAAGNAV
VLKPSEFAPSTAAFLAANIPKYLDANAVKVVQGGAEVGEELMEHRWDKVLFTGNARVGRIIMTKAAKHL
TPVALELGSKCPCIVDCLDSKRECQVAVNRIIGAKWSTCAGQACVAIDYILVEEQFAPFLIELLKSTLKRFFT
EPEYMARILNEKHFHRLTNLLEDDQVKSSIVHGGNADPKTLWIEPTIVLNPPFDSDIMMEEIFGPLLPIITVK
KTEDCIAFLKSKPKPLAIYAFTNNEKLKQRIVAETSSGSVLFNDAIVQYGLDSVPFGGIGESGFGQYHGKYT
FELFSHRKAVVRRSLLVEFMFRYPPWDEYKMGMLRRVFRFDYVSLVLALLAFWLLGIRR

>OsALDH3-2

MAPAMVAAMGEKPKPAVVLGGMVSGLREVYESGRTKDLEWRQSQLKALIRLLTDKEEEIFAVLHDDLGK
HRGESFRDELGILVKSIKYTLQNLKKWAASERAESPLVAFPATAMVVPEPLGVVLVFSCWNLPLGLALEPLS
GAIAAGNAVVLKPSELAPSTAAFLAANIPRYLDSRAVKVVLGGPNVGEELMEHRWDKVLFTGSARIGRIIM
AKAVKHLTPVALELGSKCPCIVDWLDSKRDRQIAVNRIIGAKWSTCAGQACIAIDHVIVEERFAPILIELLKS
TLKRFMAKPGGMARILNAKHFERLSGYLEDNRVAASVVHGGYMDPKKLNIEPTLLLNPPADSDVMTEEV
FGPILPIITVKKIEDCIAYLKSKPKPIAMYAFTNNERLKRRIVEETSSGSVTFNDAVVQYALESVPFGGVGHS
GFGQYHGKYSFELFSHKKAVFKRSFLIEFMFRYPPWDERKIGTLRHVFSYNYFLLFFNLLGFRR

>AtALDH3F1

MEAMKETVEESLREMRETFASGRTRSLKWRKAQIGAIYEMVKDNEDKICNALFQDLGKLSTEAFRDELG
VVLRTATVAINCLDKWAVPKHSKLPLLFYPAKGKVISEPYGTVLVLSSWNFPISLSLDPLIGAIAAGNTVLLK
SSELSPNASAFLAKTIPAYLDTKAIKVIEGGPDVATILLQHQWDKIFFTGSPKIGRIIMAAAAQHLTPVTLELG
GKCPTIVDHTISKNIKSVVKRIAGGKWGSCNGQACISVDYVLIEKSFAPTLIDMLKPTIKSFFGENPKESGCL
SRIANKHHVQRLSRLLSDPRVQASIVYGGSIDEDKLYVEPTILLDPPLDSEIMNEEIFGPILPIITVRDIQESIGI
INTKPKPLAIYAFTNDENLKTRILSETSSGSVTFNDVMIQYMCDALPFGGVGESGIGRYHGKYSFDCFSHEK
AIMEGSLGMDLEARYPPWNNFKLTFIRLAFREAYFKLILLMLGLKR

>AtALDH3H1

MAAKKVFGSAEASNLVTELRRSFDDGVTRGYEWRVTQLKKLMIICDNHEPEIVAALRDDLGKPELESSVY
EVSLLRNSIKLALKQLKNWMAPEKAKTSLTTFPASAEIVSEPLGVVLVISAWNYPFLLSIDPVIGAISAGNAV
VLKPSELAPASSALLTKLLEQYLDPSAVRVVEGAVTETSALLEQKWDKIFYTGSSKIGRVIMAAAAKHLTP
VVLELGGKSPVVVDSDTDLKVTVRRIIVGKWGCNNGQACVSPDYILTTKEYAPKLIDAMKLELEKFYGKN
PIESKDMSRIVNSNHFDRLSKLLDEKEVSDKIVYGGEKDRENLKIAPTILLDVPLDSLIMSEEIFGPLLPILTL
NNLEESFDVIRSRPKPLAAYLFTHNKKLKERFAATVSAGGIVVNDIAVHLALHTLPFGGVGESGMGAYHG
KFSFDAFSHKKAVLYRSLFGDSAVRYPPYSRGKLRLLKALVDSNIFDLFKVLLGLA

>AtALDH3

MTKLLEINHIQTLCFAKGFSPARLNVATSPFLISRRGGGGYCSNACIPYRLKFTCYATLSAVVKEQASDFRG
KEAALLVDELRSNFNSGRTKSYEWRISQLQNIARMIDEKEKCITEALYQDLSKPELEAFLAEISNTKSSCML

AIKELKNWMAPETVKTSVTTFPSSAQIVSEPLGVVLVISAWNFPFLLSVEPVIGAIAAGNAVVLKPSEIAPAA
SSLLAKLFSEYLDNTTIRVIEGGVPETTALLDQKWDKIFFTGGARVARIIMAAAARNLTPVVLELGGKCPAL
VDSDVNLQVAARRIIAGKWACNSGQACIGVDYVITTKDFASKLIDALKTELETFFGQNALESKDLSRIVNS
FHFKRLESMLKENGVANKIVHGGRITEDKLKISPTILLDVPEASSMMQEEIFGPLLPIITVQKIEDGFQVIRSK
SKPLAAYLFTNNKELEKQFVQDVSAGGITINDTVLHVTVKDLPFGGVGESGIGAYHGKFSYETFSHKKGV
LYRSFSGDADLRYPPYTPKKKMVLKALLSSNMFAAILAFFGFSKDS
>GmALDH3H2
MSGEETQRNVFGAETASSLVKELRDNFGKGTTRSYEWRVSQVKALLKAVVENEDQIVGALCSDLAKPPLE
TVVYEIGMFQNSCEVILKELKHWMTPEKVKTSIRTFPSSAEIVPEPLGVVLVISAWNYPILLSLDPVVGAIA
AGNAVVLKPSEIAPATSSVLAKLIEKYMDNSFVRVVEGAVDETTALLQQKWNKIFYTGNGRVGKIVMTAA
AKHLTPVVLELGGKSPVVVDSNNNLLVAARRIIAGKWGLNNGQACISPDYVITTKDYAPKLVDTLKTELES
FYGRNPLESEDLSRIVSSNHFARLSKLLNDDKVSGKIVYGGKKKKK
>ZmALDH7B6
MGAFAKEEHQFLAELGLAQRNPGAFACGAWGGSGPTVTSTSPTNNQVIAEVVEASVHDYEEGMRACFD
AAKTWMAIPAPKRGEIVRQIGDALRAKLHHLGRLVSLEMGKILPEGIGEVQEIIDMCDYAVGLSRQLNGSII
PSERPNHMMMEVWNPLGVVGVITAFNFPCAVLGWNACIALVCGNCVVWKGAPTTPLITIAMTKIVASVLE
KNNLPGAIFTSFCGGTEIGQAIALDIRIPLVSFTGSTRAGLMVQQQVSARFGKCLLELSGNNAIIVMDDADI
QLAVRSVLFAAVGTAGQRCTTCRRLILHENIYQTFLDQLVEVYKQVRIGDPLEKGTLLGPLHTPASKENFL
KGIQTIKSQGGKILFGGSAIESEGNFVQPTIVEITPSAPVVKEELFGPVLYAMKFQTLKEAIEINNSVPQGLSS
SIFTKRPDIIFKWLGPHGSDCGIVNVNIPTNGAEIGGAFGGEKATGGGREAGSDSWKQYMRRATCTINYGS
ELPLAQGINFG
>AtALDH5F1
MVIGAAARVAIGGCRKLISSHTSLLLVSSQCRQMSMDAQSVSEKLRSSGLLRTQGLIGGKWLDSYDNKTIK
VNNPATGEIIADVACMGTKETNDAIASSYEAFTSWSRLTAGERSKVLRRWYDLLIAHKEELGQLITLEQGK
PLKEAIGEVAYGASFIEYYAEEAKRVYGDIIPPNLSDRRLLVLKQPVGVVGAITPWNFPLAMITRKVGPALA
SGCTVVVKPSELTPLTALAAAELALQAGVPPGALNVVMGNAPEIGDALLTSPQVRKITFTGSTAVGKKLM
AAAAPTVKKVSLELGGNAPSIVFDDADLDVAVKGTLAAKFRNSGQTCVCANRVLVQDGIYDKFAEAFSEA
VQKLEVGDGFRDGTTQGPLINDAAVQKVETFVQDAVSKGAKIIIGGKRHSLGMTFYEPTVIRDVSDNMIM
SKEEIFGPVAPLIRFKTEEDAIRIANDTIAGLAAYIFTNSVQRSWRVFEALEYGLVGVNEGLISTEVAPFGGV
KQSGLGREGSKYGMDEYLEIKYVCLGDMNRH
>OsALDH2-1
MAAANGGDSKGFEVPKLEIKFTKLFINGRFVDAVSGKTFETRDPRTGEVIAKIAEGDKADIDLAVKAAREA
FDHGPWPRMSGFARGRILHKFADLVEQHVEELAALDTVDAGKLFAMGKLVDIPGGANLLRYYAGAADK
VHGETLKMARPCHGYTLKEPVGVVGHIVPWNYPTTMFFFKASPALAAGCTMVVKPAEQTPLSALFYAHL
AKLAGVPDGVLNVVPGFGPTAGAAISSHMDIDKVSFTGSTEVGRLVMEAAAKSNLKPVSLELGGKSPVIV
FDDADLDTAVNLVHMASYTNKGEICVAGSRIYVQEGIYDAFVKKATEMAKKSVVGDPFNPRVHQGPQID
KEQYEKILKYIDIGKREGATLVTGGKPCGENGYYIEPTIFTDVKEEMSIAQEEIFGPVMALMKFKTVEEAIQ
KANSTRYGLAAGIVTKNIDVANTVSRSIRAGAIWINCYLGFDPDVPFGGYKMSGFGKDMGMDALEKYLH
TKAVVTPLYNTPWL
>OsALDH5
MAMAMAMRRAAALGARHILAASSTSSSGVLLRRHMSVDAGAAMEKVRAAGLLRTQGLIGGKWVDAY
DGKTIEVQNPATGETLANVSCMGSKETSDAIASAHSTFYSWSKLTANERSKALRKWHDLIISHKEELALLM
TLEQGKPMKEALVEVTYGASFIEYFAEEAKRIYGDIIPPTLSDRRLLVLKQPVGVVGAVTPWNFPLAMITR
KVGPALACGCTVVVKPSEFTPLTALAAADLALQAGIPAGAINVVMGNAPEIGDALLQSTQVRKITFTGSTA

VGKKLMAGSANTVKKVSLELGGNAPCIVFDDADIDVAIKGSLAAKFRNSGQTCVCANRILVQEGIYEKFA
SAFIKAVQSLKVGNGLEESTSQGPLINEAAVQKVEKFINDATSKGANIMLGGKRHSLGMSFYEPTVVGNVS
NDMLLFREEVFGPVAPLVPFKTEEDAIRMANDTNAGLAAYIFTKSIPRSWRVSEALEYGLVGVNEGIVSTE
VAPFGGVKQSGLGREGSKYGMDEYLELKYICMGNLN

>OsALDH6

MLRAALLRSGSGLRRPPMAAPLSTAAAASWLSDSASSPPRVRLLIGGEFVESRADEHVDVTNPATQEVVSR
IPLTTADEFRAAVDAARTAFPGWRNTPVTTRQRIMLKYQELIRANMDKLAENITTEQGKTLKDAWGDVFR
GLEVVEHACGMGTLQMGEYVSNVSNGIDTFSIREPLGVCAGICPFNFPAMIPLWMFPIAVTCGNTFVLKPS
EKDPGAAMMLAELAMEAGLPKGVLNIVHGTHDVVNNICDDEDIKAVSFVGSNIAGMHIYSRASAKGKRV
QSNMGAKNHAIILPDADRDATLNALIAAGFGAAGQRCMALSTAVFVGGSEPWEDELVKRASSLVVNSGM
ASDADLGPVISKQAKERICKLIQSGADNGARVLLDGRDIVVPNFENGNFVGPTLLADVKSEMECYKEEIFG
PVLLLMKAESLDDAIQIVNRNKYGNGASIFTTSGVSARKFQTDIEAGQVGINVPIPVPLPFFSFTGSKASFA
GDLNFYGKAGVQFFTQIKTVTQQWKESPAQRVSLSMPTSQK

>OsALDH7

MGSFARKEHQFLAELGLAPRNPGSFACGAWGGSGPVVTSTNPTNNQVIAEVVEASAREYEEGMRACYDA
AKTWMAIPAPKRGEIVRQIGDALRAKLHHLGRLVSLEMGKILPEGIGEVQEIIDMCDYAVGLSRQLNGSIIP
SERPNHMMMEVWNPLGVVGVITAFNFPCAVLGWNACIALVCGNCVVWKGAPTTPLITIAMTKIVASVLE
RNNLPGSIFTAFCGGADIGQAISLDTRIPLVSFTGSTKVGLMVQQQVNARFGKCLLELSGNNAIIVMDDADI
QLAVRSVLFAAVGTAGQRCTTCRRLLLHESIYRTFLDQLVEVYKQVRIGDPLENGTLLGPLHTPASRDAFL
KGIQTIRSQGGKILYGGSAIESEGNFVQPTIVEISPSAPVVREELFGPVLYVMKVQNLKEAVEINNSVPQGLS
SSIFTKRPDIIFKWIGPHGSDCGIVNVNIPTNGAEIGGAFGGEKATGGGREAGSDSWKQYMRRATCTINYGS
ELPLAQGINFG

>OsALDH10-1

MAAPSAIPRRGLFIGGGWREPSLGRRLPVVNPATEATIGDIPAATAEDVELAVSAARDAFGRDGGRHWSRA
PGAVRAKYLKAIAAKIKDKKSYLALLETLDSGKPLDEAAGDMEDVAACFEYYADLAEALDGKQRAPISLP
MENFESYVLKEPIGVVGLITPWNYPLLMATWKVAPALAAGCTAVLKPSELASLTCLELGGICAEIGLPPGVL
NIITGLGTEAGAPLASHPHVDKIAFTGSTETGKRIMITASQMVKPVSLELGGKSPLIVFDDVDIDKAVEWAM
FGCFANAGQVCSATSRLLLHEKIAKRFLDRLVAWAKSIKISDPLEEGCRLGSVVSEGQYQKIMKFISTARCE
GATILYGGARPQHLKRGFFIEPTIITNVSTSMQIWREEVFGPVICVKEFRTEREAVELANDTHYGLAGAVISN
DLERCERISKAIQSGIVWINCSQPCFVQAPWGGNKRSGFGRELGQWGLDNYLSVKQVTKYCSDEPYGWY
RPPSKL

>OsALDH11

MAAVAGTGVFAEILEGEVYRYYADGEWRVSASGKSVAIVNPTTRLTQYRVQACTQEEVNKVMETAKVAQ
KAWARTPLWKRAELLHKAAAILKEHKTPIAECLVKEIAKPAKDAISEVVRSGDLVSYTAEEGVRILGEGKL
LVSDSFPGNERNKYCLSSKVPLGVVLAIPPFNYPVNLAVSKIGPALIAGNALVLKPPTQGAVAALHMVHCF
HLAGFPKGLINCVTGKGSEIGDFLTMHPGVNCISFTGGDTGIAISKKAGMVPLQMELGGKDACVVLEDAD
LDLVAANIVKGGFSYSGQRCTAVKVVLIMESVADIVVEKVKAKLAKLTVGPPEADSDITPVVTESSANFIEG
LVMDAKEKGATFCQEYRREGNLIWPLLLDHVRPDMRIAWEEPFGPVLPVIRINSVEEGIHHCNASNFGLQG
CVFTKDINKAIMISDAMETGTVQINSAPARGPDHFPFQGLKDSGIGSQGITNSINMMTKVKSTVINLPSPSY
TMG

>OsALDH12

AAAEKRRRYGGGCGDXGVRGDPGRGKCTGTTPTGXWRVSASGKSVAIVNPTXRLTQYRVQACTQEEVN
KVMETAKVAQKAWARTPLWKRAELLHKAAAILKEHKTPIAECLVKEIAKPAKDAISEVVRSGDLVSYTAE
EGVRILGEGKLLVSDSFPGNERNKYCLSSKVPLGVVLAIPPFNYPVNLAVSKIGPALIAGNALVLKPPTQGA

VAALHMVHCFHLAGFPKGLINCVTGKGSEIGDFLTMHPGVNCISFTGGDTGIAISKKAGMVPLQMELGGK
DACVVLEDADLDLVAANIVKGGFSYSGQRCTAVKVVLIMESVADIVVEKVKAKLAKLTVGPPEADSDITP
VVTESSANFIEGLVMDAKEKGATFCQEYRREGNLIWPLLLDHVRPDMRIAWEEPFGPVLPVIRINSVEEGIH
HCNASNFGLQGCVFTKDINKAIMISDAMETGTVQINSAPARGPDHFPFQGLKDSGIGSQGITNSINMMTKV
KSTVINLPSPSYTMG

>OsALDH18-1

MGRGGIGGAGLVAAVAKADVENTDSTRGFVKDVKRIIIKVGTAVVTGPNGRLAMGRLGALCEQVKQLNF
EGYEVILVTSGAVGVGRQRLKYRKLVNSSFADLQNPQMDMDGKACAAVGQSVLMAIYDTLFSQLDVTSS
QLLVTDRDFMDPSFGNQLRETVNSLLDLKVIPVFNENDAISTRRQPYEDSSGIFWDNDSLARLLAQELKAD
LLIMLSDVEGLYSGPPSDPQSKIIHTYVHEQHGKLISFGEKSRVGRGGMQAKVAAAFTASSKGIPVVIASGF
AIDSIIKVMRGEKIGTLFHREANQWGCSKEATAREMAVAARDCSRHLQKLSSEERKKILLDIADALEANED
LITSENQADLDLAQDIGYDKSLVARMTIKPGKIKSLAGSIREIADMEDPISHTLKRTEVAKDLVFEKTYCPLG
VLLIIFESRPDALVQIASLAIRSGNGLLLKGGKEAMRSNTILHKVITGAIPDVVGKKLIGLVKNKDEIADLLK
LDDVIDLVIPRGSNKLVSQIKAATKIPVLGHADGICHVYIDKSADMDMAKRIVLDAKVDYPAACNAMETL
LVHKDLNRTEGLDDLLVELEKEGVVIYGGPVAHDTLKLPKVDSFHHEYNSMACTLEFVDDVQSAIDHINR
YGSAHTDCIITTDGKAAETFLQQVDSAAVFHNASTRFCDGARFGLGAEVGISTGRIHARGPVGVDGLLTTR
CILRGSGQVVNGDKGVVYTHRELPLQ
